# Process and mechanism of preparing metallized blast furnace burden from metallurgical dust and sludge

**DOI:** 10.1038/s41598-024-60425-8

**Published:** 2024-04-29

**Authors:** Xing Gao, Yifan Chai, Yici Wang, Guoping Luo, Shengli An, Jun Peng

**Affiliations:** 1 School of Metallurgical Future Technology, Inner Mongolia University of Science and Technology, Baotou, 014000 China; 2 Key Laboratory of Green Extraction & Efficient Utilization of Light Rare-Earth Resources (Inner Mongolia University of Science and Technology), Ministry of Education, Baotou, 014010 China

**Keywords:** Metallized pellets, Dezincification rate, Metallization rate, Metallurgical dust and sludge, Pollution remediation, Sustainability

## Abstract

Metallurgical dust and sludge are solid waste resources with recycling value. In recent years, rotary hearth furnace has become the most important means to treat metallurgical dust and sludge because of its wide range of raw materials and strong treatment capacity. In this study blast furnace ash and converter sludge were selected as the research objects, and high-quality metallized pellets were prepared based on the rotary hearth furnace process. The strength changed of pellets, the reduction process of iron oxides and the removal process of zinc during the roasting of pellets in rotary hearth furnace were studied. To explore the reasonable roasting condition for preparing metallized pellets in rotary hearth furnace. The optimum roasting temperature of the pellets was 1250℃ and the roasting time was 25 min. The compressive strength, metallization rate and dezincification rate of metallized pellets reached 1361N, 97.44% and 95.67%, respectively. The efficient resource utilization of various metallurgical dust and sludge is realized.

## Introduction

In 2022, the output of crude steel in China was 1.018 billion tons^1^. It accounted for 54.01% of the global crude steel output^2^. The development of iron and steel industry has greatly promoted the development of social economy^3^. However, with the development of iron and steel industry, all kinds of metallurgical dust and sludge have been produced in the process of ironmaking and steelmaking^4–6^. According to the statistics, the amount of dust and sludge generated by iron and steel enterprises annually accounts for about 8–12% of the crude steel production^7^. According to this calculation, the amount of dust and sludge generated by Chinese iron and steel enterprises in 2022 was about 82–122 million tons. As a large industrial solid waste, the metallurgical dust and sludge contains harmful elements such as Zn, K and Na^8–10^. Simply stacking and burying metallurgical dust sludge not only takes up land resources, but also harms the environment^11,12^. At the same time, metallurgical dust and sludge is rich in valuable resources such as iron, carbon and zinc, which has high value of resource recycling^13,14^. More importantly, like some major industrial countries^15,16^, the Chinese government has listed zinc-containing dust and sludge as hazardous waste in the “Directory of National Hazardous Wastes” catalogue, and its resource utilization is particularly urgent.

At present, the methods of treating zinc-containing dust and sludge are: return sintering, stabilization, physical method, hydrometallurgical method and pyrometallurgy method^11,17^. Some scholars have studied the return sintering treatment of blast furnace ash. The results show that the return sintering process of blast furnace ash can replace part of coke powder, and the replacement rate is about 100%. Adding a certain amount of blast furnace ash can improve the sintering speed and utilization coefficient. However, the blast addition of blast furnace ash will reduce the quality of sinter^18–20^. The disadvantage of returning to sintering process is that harmful elements such as Zn will enter the blast furnace with sinter. It increases the zinc load of blast furnace. Some scholars have studied the stabilization treatment technology of metallurgical dust and sludge. The results show that it can be used to prepare stabilized products such as cement and ceramics. The advantage of this process is low energy consumption. The disadvantage is that harmful elements such as Zn exist in stabilized products and there are potential hazards to the environment^21–23^. Cao et al.^24^ studied the treatment of blast furnace gas sludge by water conservancy cyclone separation technology in Baosteel. The results show that the recovery rate can reach more than 60% under the dezincification rate of 70%. Many scholars have studied the hydrometallurgical process. Oustadakis et al.^25^ used hydrometallurgical method to treat electric furnace dust. Under the conditions of acid normality is 3N, temperature is 60℃ and solid to liquid ratio is 10%, the zinc extraction reached 80% and the iron leaching reached 45%. Teo et al.^26^ used hydrochloric acid as leaching solution to study the effects of reaction temperature and hydrochloric acid concentration on the extraction rate of iron and zinc. The results show that with the increase of reaction temperature and hydrochloric acid concentration, the extraction rate of iron and zinc increases simultaneously. Pyrometallurgical process has become the most widely used main treatment method for metallurgical dust and sludge because of its high treatment efficiency and high variety of raw materials. Rotary hearth furnace process is the representative of pyrometallurgical process^27,28^. Daniel et al.^29^ explored the treatment scheme of blast furnace dust in UK steel plant. The results show that zinc in blast furnace dust mainly exists in the form of Zn_3-x_O_4_, which will reduce the efficiency of hydrometallurgy recovery. The blast furnace dust also contained 40% fixed carbon and more suitable for rotary hearth furnace treatment. Yang et al.^30^ studied the process of preparing metallized pellets in rotary hearth furnace. The results show that the properties of pellets are good at roasting temperature was 1270℃ and roasting time was 25 min, and the dezincification rate and metallization rate reached 82.09% and 91.29%, respectively.

However, the metallized pellets produced by rotary hearth furnace have some problems, such as low metallization rate, low dezincification rate and high pulverization rate of pellets during production^31,32^.

In this study, converter sludge and blast furnace ash slag were selected as raw materials, all of which are processed by a rotary hearth furnace enterprise in southern China. Converter sludge is the product of wet dust removal of gas in converter steelmaking process, and blast furnace ash is the product of dry dust of blast furnace bag. Because the carbon content in the converter sludge is less, it is necessary to add a certain amount of carbon as reducing agent to help the pellets be fully metallized during roasting. The compressive strength, metallization rate and dezincification rate of metallized pellets were used as evaluation indicators. The high-value metallized pellets were prepared. The effects of roasting time and roasting temperature on compressive strength, metallization rate and dezincification rate of metallized pellets have been studied. Combined with XRD (X-ray Diffraction) and SEM–EDS (Scanning electron microscopy and energy dispersive spectroscopy) detection and analysis methods, the macrostructure characteristics, microstructure characteristics and phase transformation law of metallized pellets during roasting were studied. The mechanism of strength change of metallized pellets and mechanism of zinc removal were found out. The reasonable roasting condition for preparing metallized pellets in rotary hearth furnace was obtained. This study can provide theoretical guidance and technical support for the industrial production of metallurgical dust and sludge.

## Experiment

### Experimental materials

The blast furnace ash and converter sludge were selected as main raw materials. The composition of the two samples is shown in Table 1. The C and Zn content of blast furnace ash are higher, reached 17.64% and 3.19%. The TFe (Total Fe) content of converter sludge was high, reached 54.59%. The result of proximate analysis of CDQ powder (powder of coke dry quenching) is shown in Table 2.

**Table 1:** Main composition of two kinds of metallurgical dust and sludge, wt%.

Composition	TFe	Fe_2_O_3_	CaO	SiO_2_	Al_2_O_3_	MgO	SO_3_	MnO	ZnO	P_2_O_5_	C
Blast furnace ash	48.99	69.98	5.36	8.11	4.53	0.93	2.40	0.16	3.19	0.23	17.64
Converter sludge	54.59	77.99	11.00	1.14	0.29	2.38	0.46	2.38	2.68	0.15	1.09

**Table 2:** Proximate analysis of CDQ powder, wt% (dry basis).

A_d_	V_d_	FC_d_
14.50	4.10	81.40

*A*_*d*_ ash, *V*_*d*_ volatiles, *FC*_*d*_ fixed carbon.

**Figure 1:**
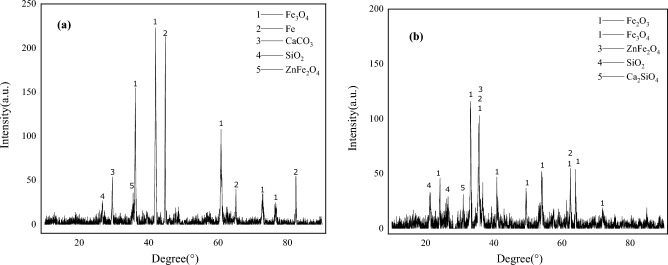
Two kinds of metallurgical dust phase diagram: (**a**) converter sludge; (**b**) Blast furnace ash;

The XRD phase analysis of two kinds of metallurgical dust sludge is shown in Fig. 1. The main phases of converter sludge are Fe and Fe_3_O_4_, and a small amount of CaCO_3_, SiO_2_ and ZnFe_2_O_4_. The diffraction peaks of Fe and FeO are high, it means that the roasting process of pellets prepared with converter sludge as the main raw material is more conducive to the reduction of iron oxides and the reduction of iron oxides will consume less carbon. Zn mainly exists in the form of ZnFe_2_O_4_ in converter sludge. The main phases of blast furnace ash are Fe_2_O_3_ and ZnFe_2_O_4_, and a small amount of SiO_2_ and Ca_2_SiO_4_. The diffraction peak is about 20 degrees and there are a lot of miscellaneous peaks, which are irregular diffraction peaks of C. Compared with converter sludge, it can be clearly felt that the carbon content in blast furnace ash is much higher than that in converter sludge. Zinc mainly exists in the form of ZnFe_2_O_4_ in blast furnace ash. The XRD phase and XRF detection results of two kinds of metallurgical dust and sludge are consistent.

### Preparation of metallized pellets

The converter sludge, blast furnace ash and CDQ powder were dried and ground to below 200 mesh (− 0.074 μm). The converter sludge, blast furnace ash and CDQ powder were mixed according to the C/O (Carbon/oxygen is the fixed carbon in CDQ powder and carbon in two kinds of metallurgical dust and sludge divided by oxygen in iron oxide in metallurgical dust and sludge) ratio of 0.6. 4% binder was added and mixed them evenly. The contents of raw materials in green pellets are shown in Table 3. After mixing homogeneously, the chemical composition of the raw materials for preparing metallized pellets is shown in Table 4.

**Table 3:** Contents of different raw materials, wt%.

Blast furnace ash	Converter sludge	Binder	CDQ power
26.0	70	4.0	5.5

**Table 4:** Chemical composition of the raw materials for preparing metallized pellets, wt%.

Fe_3_O_4_	CaO	SiO_2_	Al_2_O_3_	MgO	SO_3_	MnO	ZnFe_2_O_4_	P_2_O_5_	C
70.03	8.96	3.02	1.50	1.88	0.94	1.68	7.91	0.16	9.83

**Figure 2:**
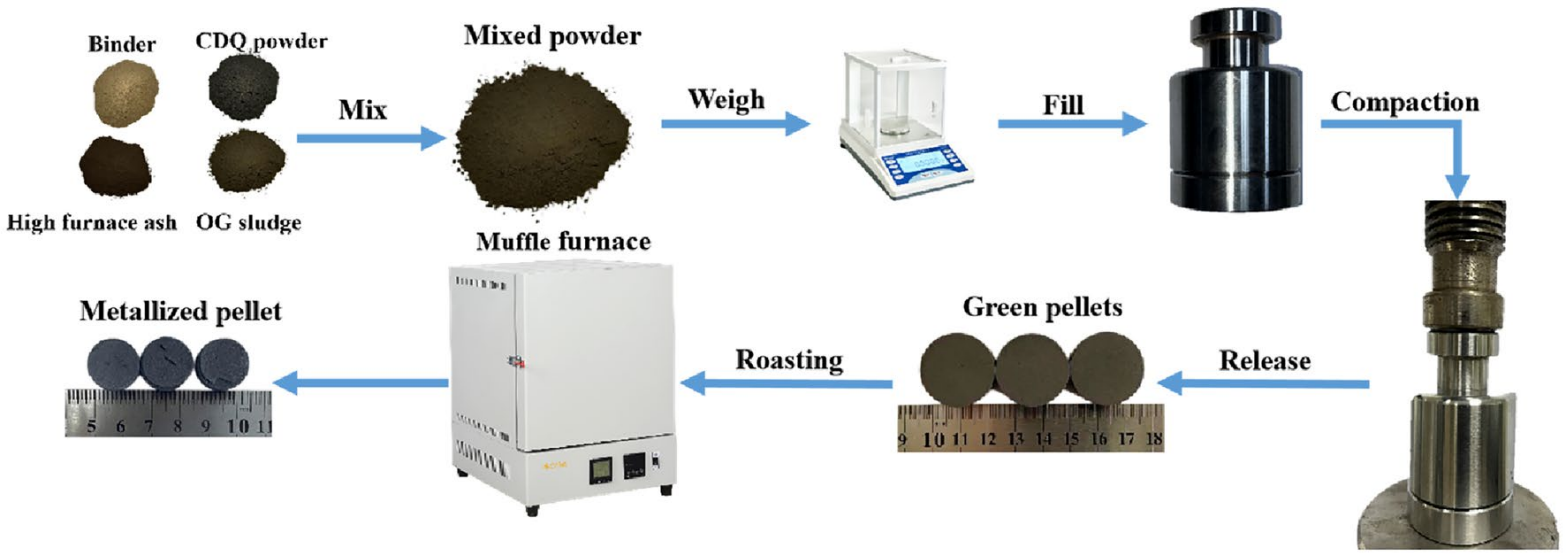
Preparation process of metallized pellets.

The preparation process of metallized pellets is shown in Fig. 2. From Fig. 2, a total of 200 g raw materials were weighed and mixed for 2 h, each time. The well-mixed samples were weighed, and each sample was weighed 25 g. The samples were filled in the mold, and then put on the pressure head. The mold was placed on a preforming machine (the pressure was 25 Mpa and the holding time was 5 min). The green pellets were obtained. The metallized pellets were roasted by muffle furnace. The dry pellets were put in a corundum crucible with a cover. The N_2_ was introduced into the crucible and the crucible cover was quickly covered. The air in that crucible was exhaust. The crucible filled with pellets was placed in a muffle furnace with a temperature of 200 °C. The crucible filled with pellets was preheated in muffle furnace for 20 min. The preheated crucible filled with pellets was put into a muffle furnace which has been raised to a predetermined roasting temperature.

### Experimental index

Metallization rate and dezincification rate are the important index in research. The contents of Zn, Fe(TFe) and metallic iron(MFe) in the metallized pellets calcined under different roasting conditions were determined. The rate of metallization and dezincification was calculated as follows:

1 $$ {\text{M}} = \frac{{MF{\text{e}}}}{{TF{\text{e}}}} \times 100\% $$

2 $$ {\text{D}} = \frac{{{\text{m}}_{1} \times {\text{x}}_{1} - {\text{m}}_{2} \times {\text{x}}_{2} }}{{{\text{m}}_{1} \times {\text{x}}_{1} }} \times 100\% $$

*M* represents the metallization rate, %; *D* represents the dezincification rate, %; *TFe* represents the total iron content in metallized pellets, %; *MFe* represents the metallic iron content in metallized pellets, %; *m*_*1*_ represents the total mass of pellets before reduction, g; *m*_*2*_ represents the total mass of metallized pellets, g; *x*_*1*_ represents the Zn content in dry pellets, %; *x*_*2*_ represents the Zn content in metallized pellets, %.

### Detection method

The compositions of the two kinds of metallurgical dust and sludge were determined by professional institutions (Analysis and testing center of University of Science and Technology Beijing, China) through X-ray Fluorescence Spectrometer (XRF). The proximate analysis of CDQ powder was measured according to ISO 17246-2005(E). The MFe (metallic iron) and TFe content in the metallized pellets was determined according to the international standards ISO 5416:2006 and ISO 2597-2:2019. The compressive strength of metallized pellets was measured by Microcomputer Controlled Electronic Universal Testing Machine (WDW-20). The zinc content of the pellets before and after roasting was determined using an inductively coupled plasma emission spectrometer (ICP-OES). The phase composition of metallized pellets was analyzed by XRD (XTALAB SYNERGY-ED) (40 kV, 40 mA, wavelength 0.154 nm). The scanning angle range is from 10° to 90°, and the scanning speed is 0.8°/s. SEM–EDS was used to observe and detect the morphology and composition of the metallized pellets.

## Results and discussion

### Macroscopic morphology of metallized pellets

**Figure 3:**
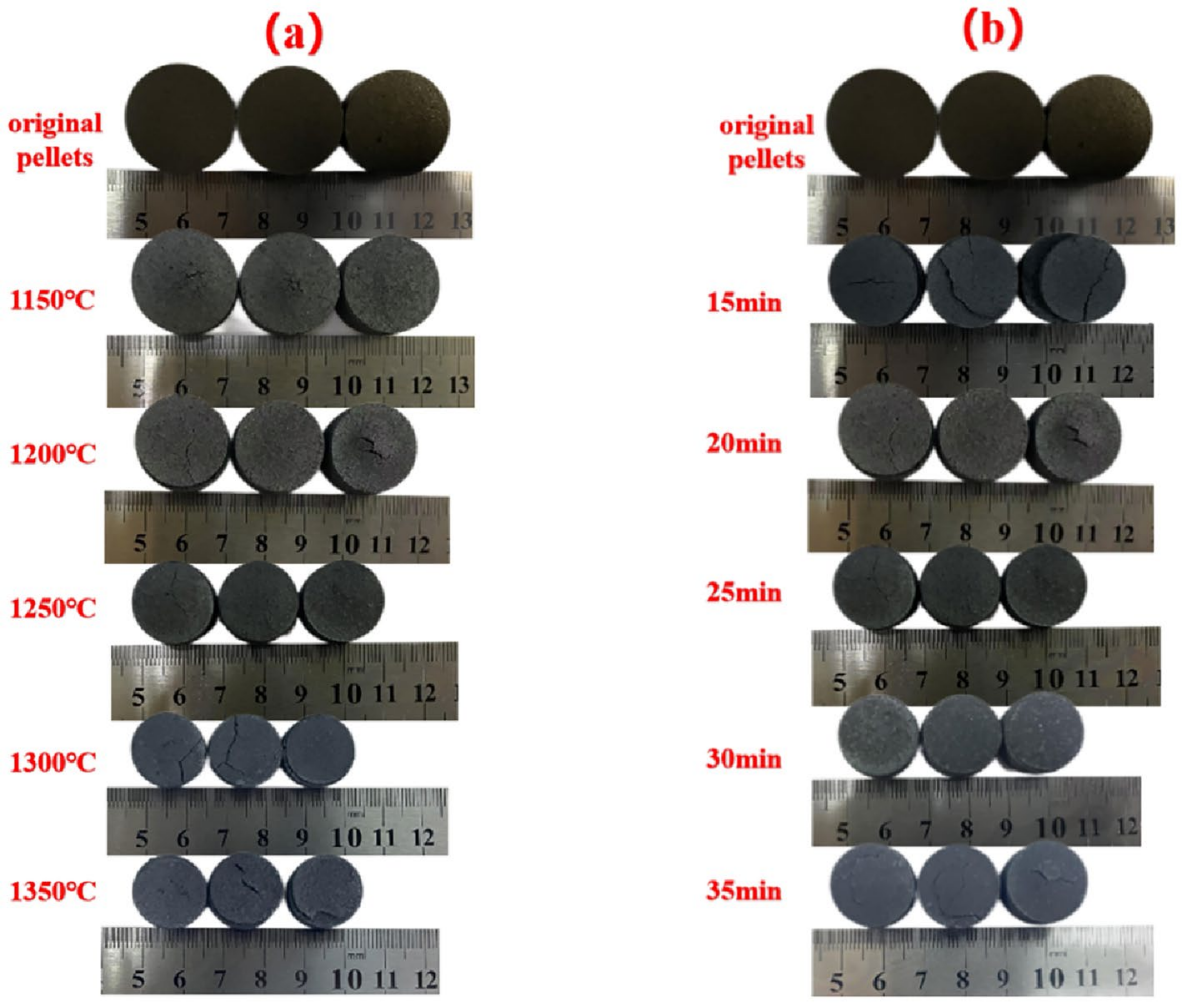
Physical pictures of metallized pellets with different roasting conditions: (**a**) different roasting temperature; (**b**) different roasting time.

The macroscopic morphology of metallized pellets roasted by muffle furnace under different roasting conditions were shown in Fig. 3. Figure 3a shows the metallized pellets with different roasting temperatures and roasting time of 25 min. Figure 3b shows the metallized pellets with different roasting time and roasting temperature of 1250 °C. It can be seen from Fig. 3 that with the increase of roasting temperature and time, the volume of metallized pellets has obviously shrunk. This is due to the precipitation of carbon in pellets and the formation of iron crystals. After the roasting temperature of 1300 °C in Fig. 3a and the roasting time of 25 min in Fig. 3b, the pellet volume shrinkage was not obvious. The reaction temperature or time was insufficient, the reduction reaction in the pellet was not fully carried out, and the iron crystal was not sufficient. Comparing Fig. 3a and b, the volume of pellets with roasting temperature above 1300 °C is smaller than that with roasting temperature of 1250 °C. However, under the conditions of roasting temperature of 1250 °C and roasting time of 25 min, the pellet volume shrinkage is not obvious. when roasting time was prolonged. It shows that the internal reduction reaction of pellets has been basically completed at roasting temperature of 1250 °C and roasting time of 25 min. The smaller pellet volume at 1300 °C and 1350 °C was due to the higher roasting temperature. It makes the reduction reaction inside the pellets more intense and the pellets have fewer pores. Therefore, from the macroscopic morphology of pellets, the reduction reaction in pellets can only be fully carried out when the roasting temperature is 1250 °C and the roasting time is 25 min. Under this condition, the pellet has a high degree of iron crystallization and high compressive strength.

### Effect of roasting conditions on compressive strength of pellets

**Figure 4:**
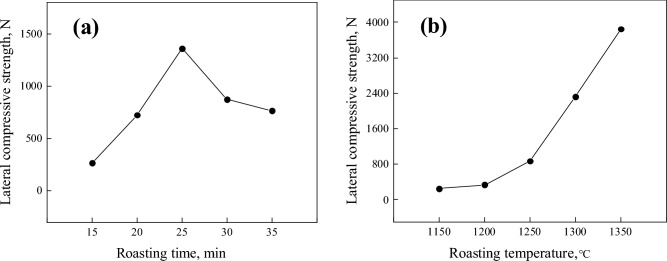
Lateral compressive strength of pellets with different roasting conditions: (**a**) different roasting time; (**b**) different roasting temperature.

In Fig. 4a, the lateral compressive strength of metallized pellets was first increased and then decreased with the extension of roasting time from 15 to 35 min. When the roasting time was 25 min, it reached the highest value of 1361 N. Before the roasting time of 25 min, the internal reduction reaction of pellets was not fully carried out. At this time, the carbon in the pellet could not be consumed completely. The insufficient iron crystals and residual carbon particles will hinder the strength of pellets. With the extension of roasting time, carbon particles were completely consumed. Iron crystals grow further, and the pores left by the precipitation of carbon gradually decrease due to the continuous crystallization of iron. This greatly increased the compressive strength of pellets. Continue to prolong the roasting time, and the lateral compressive strength of pellets will decrease. This is due to the complete consumption of carbon in pellets. Moreover, a weakly oxidizing atmosphere appeared on the pellet surface, and an iron oxide shell appeared on the outer surface of the pellet. It makes the compressive strength of pellets decrease.

It can be seen from Fig. 4b that the lateral compressive strength of metallized pellets increases with the increase of roasting temperature. The increment of lateral compressive strength is very small before 1250 °C. But the lateral compressive strength of metallized pellets increases greatly after 1250 °C. This is because the melting temperature of some gangue components such as FeO, CaO and SiO_2_ between 1150 and 1250 °C has not yet reached, and a sufficient amount of liquid has not been generated. After 1250 °C, with the increase of roasting temperature, the degree of iron connection of pellets became higher and higher. At the same time, the gangue components in the pellet formed liquid slag to fill the gaps in the pellet and improve the internal heat transfer. This increased the compressive strength of pellets.

### Effect of roasting conditions on metallization rate and dezincification rate of pellets

The results of metallization rate and dezincification rate of metallized pellets are shown in the Table 5. The highest metallization rate of pellets is 97.44%, and its corresponding roasting time and roasting temperature are 25 min and 1250 °C.

**Table 5:** Metallization and dezincification rates of metallized pellets.

Code	Roasting temperature (°C)	Roasting time (min)	Metallization ratio (%)	Dezincification rate (%)
1	1250	15	89.23	77.63
2	1250	20	94.42	93.15
3	1250	25	97.44	95.67
4	1250	30	94.04	96.51
5	1250	35	93.34	98.08
6	1150	25	83.86	43.34
7	1200	25	93.19	81.44
8	1300	25	93.10	96.09
9	1350	25	90.87	96.81

**Figure 5:**
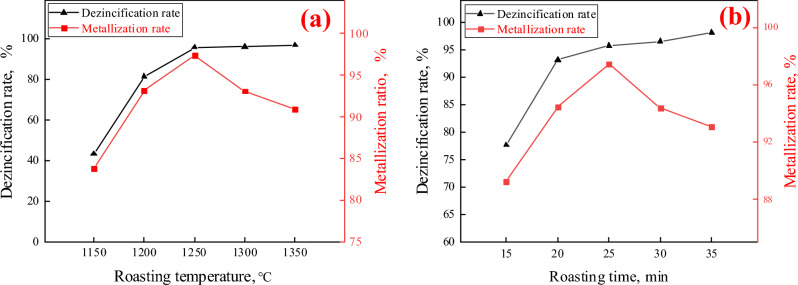
Dezincification and metallization rates of metallized pellets with different roasting conditions: (**a**) different roasting time; (**b**) different roasting temperature.

As can be seen from Fig. 5a, the metalized pellet dezincification rate increased with the increase of roasting temperature from 1150 to 1350 °C. The dezincification rate of the pellet reached more than 95% after the roasting temperature reached 1250 °C. It meets product requirements. With the increase of roasting temperature, the pellet metallization rate appeared to increase and then decrease. This is due to the fact that the pellet reduction reaction was not completed with the roasting time less than 25 min and the roasting temperature is 1250 °C. The kinetic conditions for the reduction of iron oxides within the pellet are more favorable and the reduction rate is faster with the increase of the roasting temperature after 1250 °C. This made the pellet to be restored in 25 min. The roasting temperature continued to increase and the reduction reaction within the pellet occurred too violently. The carbon inside the pellet has been completely consumed with the roasting time less than 25 min. A Weakly oxidizing atmosphere appeared on the surface of pellets, which reduces the metallization rate of pellets.

In Fig. 5b, the metallization rate of metallized pellets increased and then decreased when the roasting time was extended from 15 to 35 min at a roasting temperature of 1250 °C. The metallization rate of metallized pellets with roasting time of 25 min reached 97.44% at the highest. The lowest metallization rate of 89.23% was achieved for 15 min metallization pellets with roasting time. The metallization rate increased before the roasting time of 25 min and prolonged roasting time. The metallization rate decreases by prolonging the roasting time after 25 min. This is due to the fact that the reduction reaction of the metallized pellets was reacted within 25 min. After that continue to extend the roasting time pellets due to the equipment and equipment of the cause of re-oxidation occurs to lead to the reduction of the rate of metallization of the pellets. The roasting time was extended from 15 to 35 min, and the pellet dezincification rate continued to increase. And the dezincification rate of pellet reached more than 95% after 25 min. The zinc content in metallized pellets is 0.126% at roasting temperature of 1250 °C and roasting time of 25 min.

The metallization and dezincification rates showed different trends after 25 min. This is due to the competitive mechanism for the reduction of oxides of Fe and Zn. Carbon was more plentiful in the early stages of the roasting reaction and both were reduced at the same time. After 25 min, a weakly oxidizing atmosphere appeared around the pellets. The re-oxidation of metallic iron made the metallization rate decrease, while zinc has become zinc vapor and volatilized from the pellets, so the dezincification rate has not decreased.

### Effect of roasting conditions on XRD phase of pellets

**Figure 6:**
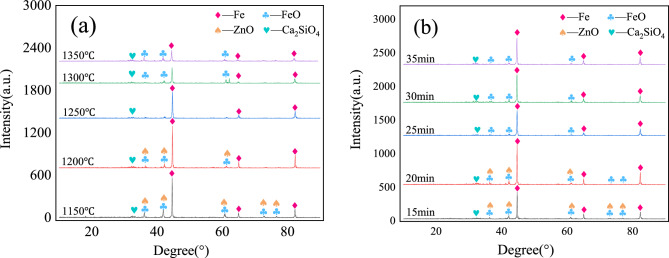
X-ray Diffraction phase diagram of pellets with different roasting conditions: (**a**) The roasting time is 25 min; (**b**) The roasting temperature is 1250 °C.

The phase diagram of metallized pellets roasted by different roasting conditions is shown in Fig. 6. The main phase of metallized pellets is Fe with a small amount of FeO, ZnO, and Ca_2_SiO_4_. The greater the intensity of diffraction peak, the better the degree of crystallization and the larger the grain size. Its corresponding crystal growth is also orderly. With the increase of roasting temperature, the diffraction peak intensity of the main phase Fe increases first and then decreases. This is because the pellets were put into the furnace and heated rapidly. The higher the temperature, the better the kinetic conditions for reduction and the faster the reduction rate. When the roasting temperature is above 1200 °C, the reduction speed in pellets is too fast, and the crystallization effect becomes worse, which is not conducive to the crystal growth in one direction. However, X-ray Diffraction can only scan the crystal in the same direction, so the diffraction peak of pellet iron crystal decreases with the increase of roasting temperature. It can be seen from Fig. 6b that the phase of ZnO gradually decreases with the extension of roasting time, and the phase of ZnO basically disappears after roasting time of 25 min, which proves that the dezincification rate is high at this time.

### Effect of roasting conditions on microstructure of pellets

**Figure 7:**
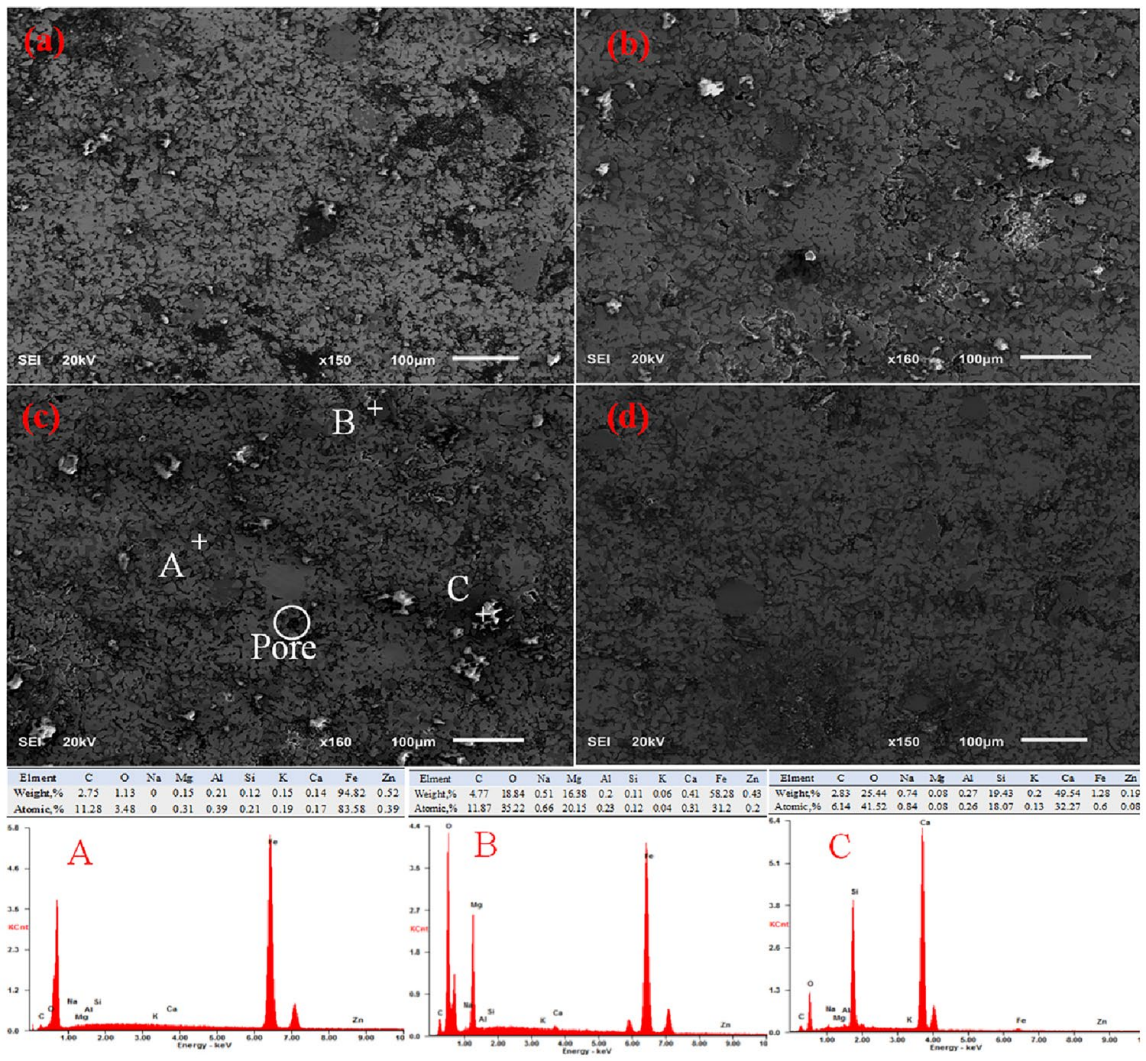
SEM–EDS morphology of pellets with different roasting times under roasting temperature of 1250 °C. (**a**): The roasting time is 15 min; (**b**): The roasting time is 20min; (**c**): The roasting time is 25 min; (**d**): The roasting time is 30 min.

The internal microstructure of metallized pellets roasted at 1250 °C for different roasting time are shown in Fig. 7a–d. Figure 7c was selected for EDS dot energy spectrum analysis, and the result is shown in Fig. 7. The metallized pellets mainly include three phases, the phase of light gray part is metallic iron phase, and its main element is Fe; The phase of dark gray part is spinel phase, and its main elements are Fe, Mg and O; The phase of bright white part is gangue phase (slag phase), and its main elements are Ca, Si and O. The dark black parts are pores. It can be clearly seen that the slag phase mainly appeared in the pores, so the low melting point liquid phase filled part of the pores during roasting, thus improving the strength of pellets. With the extension of roasting time, the porosity of metallized pellets decreases, the iron crystals were arranged more closely, and the slag phase decreases. This is because the reduction reaction was carried out in the pellet with the extension of roasting time, and the iron crystal of the pellet was denser.

### Evolution mechanism of pellet strength and dezincification mechanism

**Figure 8:**
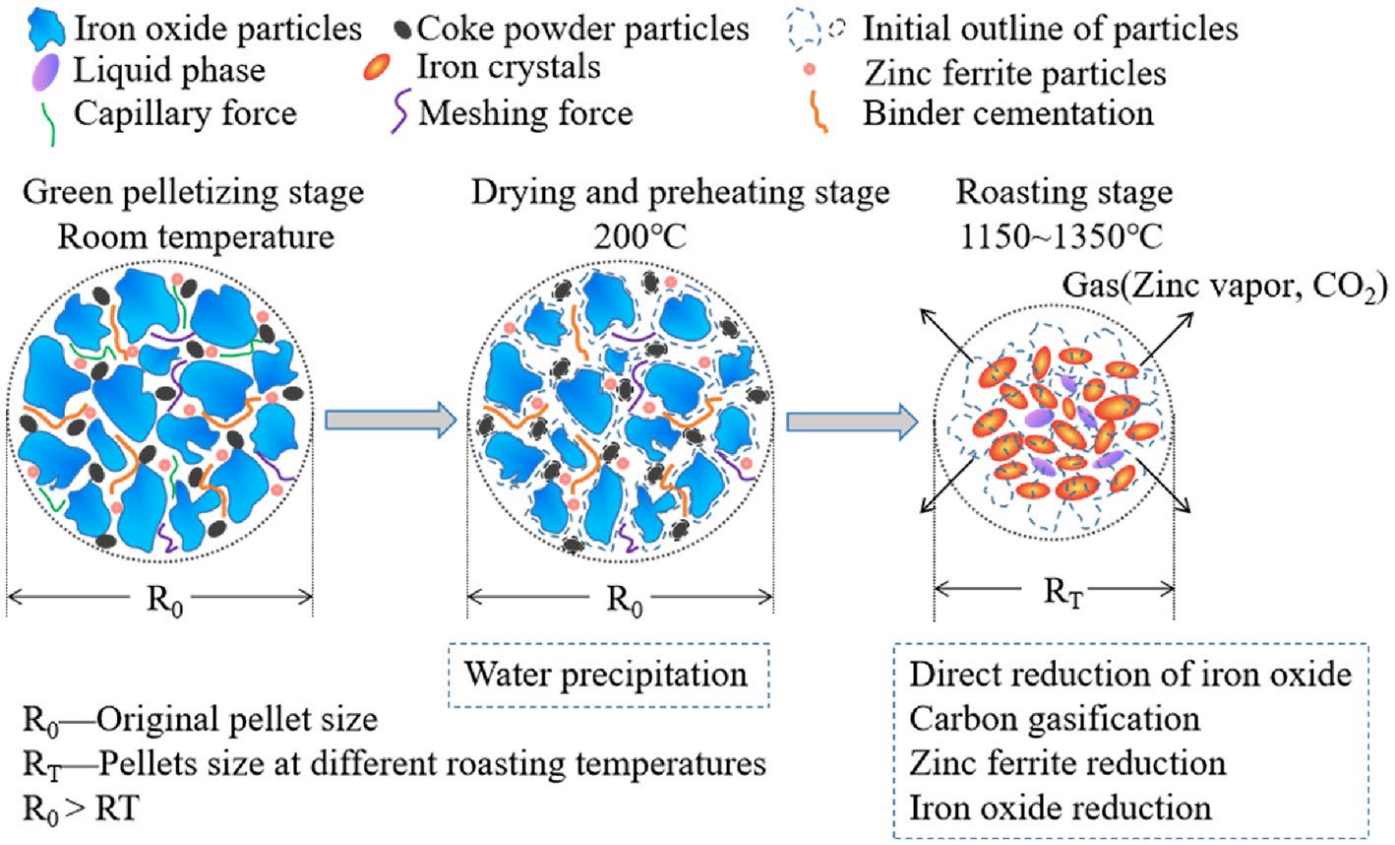
Evolution mechanism of pellet strength and dezincification mechanism.

The evolution mechanism of pellet strength and dezincification mechanism is shown in Fig. 8. The strength of green pellets is mainly composed of capillary force of water, binder cementation force of binder and meshing force of extrusion molding. With the progress of drying and preheating, the moisture in pellets was evaporated. The capillary force of water was weakened and the binder cementation force of binder was enhanced. In the roasting stage, high temperature made the binder cementation force ineffective. At the same temperature, the precipitation of carbon made the interior of pellets become porous and loose. This made the meshing force of pellets disappear. The reduction of iron oxide made the continuous crystal of iron appear, and the strength of pellets is mainly metallic bonds and electrovalent bonds. At the same time, some low melting point liquid phases were gradually formed at high temperature. These filled the internal pores of pellets and further improved the strength of pellets. At the roasting temperature of 1150 °C, the binder cementation force of pellet was disappeared, but the degree of iron crystallization was insufficient. Therefore, the compressive strength of pellets was low at this time. With the increased of roasting temperature, the reduction reaction efficiency of pellets increased gradually. At this time, a large number of iron crystals were generated, and some liquid phases were also generated to fill the internal pores of the pellets. These increased the strength of the pellets. The main occurrence form of Zn in green pellets was zinc ferrite. With the roasting, zinc ferrite was reduced by C and CO generated by gasification reaction in the pellets, and zinc vapor was generated and volatilized out of the pellet.

## Conclusion

In summary, the effect of roasting conditions on metallurgical properties of metallized pellets is studied. Novel process parameters for preparing metallized pellets with high strength, high metallization rate and high dezincification rate were obtained. The efficient and comprehensive utilization of converter sludge and blast furnace ash has been realized.

1. With the extension of roasting time, the strength of pellets first increased and then decreased. The maximum compressive strength of pellets is 1361N under the roasting time of 25 min. The compressive strength of pellets increases with the increase of roasting temperature.
2. With the increase of roasting time and roasting temperature, the metallization rate of pellets increased first and then decreased. Under the roasting time is 25 min and the roasting temperature is 1250 °C, the metallization rate of pellets can reach 97.44%, Metal Fe is the main material phase.
3. The dezincification rate of metallized pellets showed an increasing trend with the increase of roasting time and roasting temperature. The roasting temperature reached 1250 °C roasting time 25 min, the dezincification rate of the pellets can reach more than 95%. The zinc content in metallized pellets is 0.126% at roasting temperature of 1250 °C and roasting time of 25 min.
4. Compressive strength, dezincification rate, and metallization rate of the metallized pellets were considered. The optimum roasting temperature of the pellets was 1250 °C and the roasting time was 25 min. The metallization rate, dezincification rate and compressive strength of metallized pellets reached 97.44%, 95.67% and 1361N, respectively.

## Data Availability

The datasets used and analysed during the current study available from the corresponding author on reasonable request.
